# Prevalence and Molecular Detection of Carbapenemase Genes in Clinical *Klebsiella pneumoniae* Isolates From Khartoum State, Sudan

**DOI:** 10.1155/ijm/8850410

**Published:** 2026-09-17

**Authors:** Elham A. Elneel, Mahadi H. Abdallah

**Affiliations:** ^1^ Department of Microbiology, Faculty of Medical Laboratory Sciences, National University, Khartoum, Sudan, nu.edu; ^2^ Department of Microbiology, Faculty of Medical Laboratory Sciences, Alzaiem Alazhari University, Khartoum, Sudan, aau.edu.sd

**Keywords:** *bla_IMP_*, *bla_KPC_*, *bla_NDM_*, *bla_OXA-48_*, *bla_SPM_*, carbapenemase genes, carbapenem-resistant *Klebsiella pneumoniae* (CRKP)

## Abstract

**Background:**

Carbapenem‐resistant *Klebsiella pneumoniae* (CRKP) is a critical global public health threat due to its multidrug resistance and limited treatment options.

**Objective:**

This study is aimed at detecting carbapenemase genes in CRKP isolates obtained from various clinical specimens collected in Khartoum State, Sudan.

**Methods:**

Fifty CRKP isolates were collected between November 2022 and February 2023 from urine, wound swabs, high vaginal swabs, sputum, stool, and blood samples. Demographic data and antibiotic history were recorded. DNA extraction and multiplex PCR were performed to detect *bla_KPC_*, *bla_NDM_*, *bla_OXA-48_*, *bla_IMP_*, and *bla_SPM_* genes.

**Results:**

Of the 50 isolates, 44 (88%) carried at least one carbapenemase gene, whereas 6 (12%) were negative for all tested genes. The most common gene was *bla_KPC_* (72%), followed by *bla_NDM_* (42%), *bla_SPM_* (38%), *bla_IMP_* (28%), and *bla_OXA-48_* (10%). Multiple carbapenemase genes were detected in 28 isolates (56% of total isolates; 63.6% of positive isolates). No statistically significant associations were found between carbapenemase gene presence and patient age group or gender (*p* > 0.05). A significant association was observed between prior antibiotic exposure and the presence of *bla_SPM_* *(p* ≈ 0.020), and between *bla_NDM_* and infection site (*p* = 0.039), whereas no significant associations were found with the other genes.

**Conclusion:**

This study demonstrates a high prevalence of carbapenemase genes among CRKP isolates in Khartoum State, with frequent coexistence of multiple resistance genes. The significant association between prior antibiotic use and *bla_SPM_* highlights the role of antimicrobial pressure in resistance development. These findings emphasize the need for improved infection control measures and effective antimicrobial stewardship to limit the spread of carbapenem‐resistant *K. pneumoniae*.

## 1. Introduction

Carbapenem‐resistant *Klebsiella pneumoniae* (CRKP) has become a major global public health concern due to its rapid spread and the limited treatment options available. As a member of the Enterobacteriaceae family, *K. pneumoniae* is an opportunistic pathogen responsible for various hospital‐acquired infections, including pneumonia, urinary tract infections, bloodstream infections, and surgical site infections. The growing prevalence of carbapenem resistance has severely undermined the effectiveness of last‐line antibiotics like carbapenems, leading to increased mortality, longer hospital stays, and rising healthcare costs [1].

The resistance in CRKP is primarily mediated by the production of carbapenemase enzymes, which hydrolyze carbapenem antibiotics, rendering them ineffective. These enzymes are encoded by carbapenemase genes, including *bla_KPC_*, *bla_NDM_*, *bla_OXA-48_*, and *bla_VIM_*, among others [2]. Accurate detection and identification of these genes are crucial for implementing effective infection control measures and developing novel therapeutic strategies. Recent advancements in molecular methods, such as polymerase chain reaction (PCR) and whole‐genome sequencing (WGS), have significantly enhanced the ability to detect carbapenemase genes directly from clinical specimens, offering rapid and precise diagnostic capabilities [3].

The widespread presence of CRKP is largely due to the horizontal transfer of resistance genes via plasmids, which enables their dissemination across different bacterial species. This gene transfer has been documented both in hospitals and in community settings, raising serious concerns about the broader spread of resistance [4]. Compounding the issue, many CRKP strains carry more than one carbapenemase gene such as *bla_KPC_*, *bla_NDM_*, and *bla_OXA-48_*, making treatment even more difficult. These multidrug‐resistant strains often require the use of more toxic or less effective antibiotics [5, 6].

This study is aimed at investigating the molecular detection of carbapenemase genes in *K. pneumoniae* isolates from various clinical specimens. By identifying the prevalence of specific carbapenemase genes, the findings can contribute to better understanding of the epidemiology of carbapenem resistance and guide the selection of appropriate therapeutic approaches for managing CRKP infections.

## 2. Material and Methods

This cross‐sectional study analyzed 50 culture‐positive samples of phenotypically CRKP obtained from various clinical specimens, including urine, wound swabs, high vaginal swabs, sputum, stool, and blood samples. The samples were collected from patients between November 2022 and February 2023 at Baraha Medical City, Omdurman Military Hospital, and Federal Central Police Hospital in Khartoum State, Sudan.

Demographic information (age, gender, etc.), antibiotic exposure history, and infection sites were gathered using a structured questionnaire.

### 2.1. Bacterial Isolation and Identification

The clinical samples were cultured on blood agar, MacConkey agar, and chromogenic media. The cultures were incubated aerobically for 24 h at 37°C. Bacterial identification was performed based on colonial morphology, Gram staining, motility testing and standard biochemical tests, including indole, urease, and citrate tests, as per the conventional diagnostic protocols.

### 2.2. Susceptibility Testing

Antibiotic susceptibility testing for imipenem (10 *μ*g) and meropenem (10 *μ*g) was performed using the Kirby–Bauer disk diffusion method on Mueller–Hinton agar. Isolates were categorized as carbapenem‐resistant if they exhibited an inhibition zone diameter of ≤ 19 mm for imipenem or meropenem, according to the Clinical and Laboratory Standards Institute (CLSI, 2020) guidelines [7]. Only phenotypically confirmed CRKP isolates were included in this study to prevent potential selection bias and systematically exclude susceptible strains.

### 2.3. Molecular Characterization

#### 2.3.1. DNA Extraction

Total bacterial DNA was extracted from *K. pneumoniae* isolates using the following procedure: A 2‐mL tube containing 1.5 mL of bacterial culture grown in tryptophan water broth was centrifuged at 4000 rpm for 5 min. After centrifugation, the supernatant was discarded, and the bacterial pellet was resuspended in 300 *μ*L of DNase/RNase‐free water. The suspension was vortexed briefly, then heated at 95°C for 15 min. After cooling at 4°C for 5–10 min, the mixture was centrifuged again at 4000 rpm for 5 min. The supernatant containing the extracted DNA (150 *μ*L) was transferred to a new 1.5‐mL tube for further analysis. The yield and integrity of the extracted DNA were visually confirmed using 1% agarose gel electrophoresis stained with ethidium bromide prior to PCR amplification.

#### 2.3.2. PCR

The presence of the carbapenemases genes was determined using gene‐specific primer targeting *bla_NDM_, bla_IMP_, bla_SPM_, bla_KPC_*, and *bla_OXA-48_*. The primers were obtained from the Macrogen Company, Korea (Table 1) [8]. Two multiplex PCR reactions were conducted. The first multiplex reaction targeted the *bla_NDM_*, *bla_SPM_*, and *bla_IMP_* genes, whereas the second reaction focused on the *bla_KPC_* and *bla_OXA-48_* genes.

**Table 1:** Primer sequences used in this study [8].

Gene	Primer sequence	Amplicon size (bp)
*bla_KPC_*	Forward:	498
	CATTCAAGGGCTTTCTTGCTGC	
	Reverse:	
	ACGACGGCATAGTCATTTGC	

*bla_NDM_*	Forward:	624
	GGTTTGGCGATCTGGTTTTC	
	Reverse:	
	CGGAATGGCTCATCACGATC	

*bla_IMP_*	Forward:	568
	TCGTTTGAAGAAGTTAACG	
	Reverse:	
	ATGTAAGTTTCAAGAGTGATGC	

*bla_OXA-48_*	Forward:	281
	GCTTGATCGCCCTCGATT	
	Reverse:	
	GATTTGCTCCGTG GCCGAAA	

*bla_SPM_*	Forward:	271
	AAAATCTGGGTACGCAAACG	
	Reverse:	
	ACATTATCCGCTGGAACA	

In each PCR run, confirmed positive control strains harboring *bla_KPC_*, *bla_NDM_*, *bla_OXA-48_*, *bla_IMP_*, and *bla_SPM_* as well as a nuclease‐free water negative control were included to ensure reaction specificity and rule out contamination.

The first multiplex PCR reaction (targeting *bla_NDM_*, *bla_SPM_*, and *bla_IMP_*) was carried out in a total volume of 20 *μ*L containing 5 *μ*L of 2X PCR Master Mix (Maximum PCR Premix Kit i‐Taq, iNtRON Biotechnology, Korea), 0.6 *μ*L of each forward and reverse primer (10 *μ*M, totaling 3.6 *μ*L), 5 *μ*L of extracted DNA template (1–10 ng/*μ*L), and 6.4 *μ*L of nuclease‐free water. For the second multiplex reaction (targeting *bla_KPC_* and *bla_OXA-48_*), the same concentrations and volumes of primers (0.6 *μ*L each, totaling 2.4 *μ*L), DNA template (5 *μ*L), and Master Mix (5 *μ*L) were used, adjusted with 7.6 *μ*L of nuclease‐free water to reach a final reaction volume of 20 *μ*L.

PCR conditions for the first multiplex reaction were programmed in Mastercycler Eppendorf (Eppendorf, Germany) as follows: initial denaturation at 94°C for 5 min, followed by 35 cycles of denaturation at 94°C for 20 s, annealing at 56°C for 10 s, and extension at 72°C for 20 s, with a final extension at 72°C for 5 min. The same program was used for the second reaction, except the annealing temperature was set to 52°C for 30 s (Tables 2 and 3.

**Table 2:** Amplification program for *bla_NDM_*, *bla_SPM_*, and *bla_IMP_* genes.

Step	Temperature	Duration	Cycles
Initialization	94°C	5 min	1 cycle
Denaturation	94°C	20 s	35 cycles
Annealing	56°C	10 s	35 cycles
Extension/elongation	72°C	20 s	35 cycles
Final elongation	72°C	5 min	1 cycle

**Table 3:** Amplification program for *bla_OXA-48_* and *bla_KPC_* genes.

Step	Temperature	Duration	Cycles
Initialization	94°C	5 min	1 cycle
Denaturation	94°C	20 s	35 cycles
Annealing	52°C	10 s	35 cycles
Extension/elongation	72°C	20 s	35 cycles
Final elongation	72°C	5 min	1 cycle

Following amplification, 5 *μ*L of each PCR product was analyzed by electrophoresis on a 1% agarose gel prepared in 1X TAE buffer and stained with ethidium bromide (handled with proper safety precautions). Electrophoresis was conducted at 100 V for 30–45 min. The PCR products were visualized under UV light and compared with a 100 bp DNA ladder (Promega, Germany) to confirm the expected amplicon sizes: 624 bp for *bla_NDM_*, 271 bp for *bla_SPM_*, 568 bp for *bla_IMP_*, 498 bp for *bla_KPC_*, and 281 bp for *bla_OXA-48_*.

### 2.4. Ethical Considerations

Ethical approval for this study was obtained from the Ethics Committee of the Faculty of Medical Laboratory Sciences, Alzaiem Alazhari University, Khartoum, Sudan. Informed consent was waived by the ethics committee due to the retrospective nature of the study, which utilized anonymized clinical isolates without direct patient contact.

### 2.5. Statistical Analysis

Data were analyzed using SPSS Version 26.0 (IBM Corp., Armonk, New York, United States). Categorical variables were expressed as frequencies and percentages. Associations between carbapenemase gene presence and categorical parameters (infection site, age group, gender, antibiotic exposure) were evaluated using the chi‐square test or Fisher′s exact test where appropriate. A *p* value of < 0.05 was considered statistically significant. Given the exploratory nature of this cross‐sectional study and the small subgroup sizes, no formal adjustments for multiple comparisons (e.g., Bonferroni correction) were applied to the *p* values. Therefore, isolated significant associations should be interpreted with caution.

## 3. Result

This study analyzed 50 CRKP isolates from various clinical specimens. The distribution of isolates was as follows: urine samples (22 isolates, 44%), wound swabs (13 isolates, 26%), high vaginal swabs (3 isolates, 6%), sputum (6 isolates, 12%), stool (2 isolates, 4%), and blood (4 isolates, 8%). Of these, 31 isolates (62%) were from male patients and 19 (38%) from female patients. The age distribution of patients was under 15 years (8 patients, 16%), 16–40 years (14 patients, 28%), 41–65 years (14 patients, 28%), 66–90 years (13 patients, 26%), and over 90 years (1 patient, 2%). Additionally, 41 patients (82%) had a history of prior antibiotic exposure.

**Figure 1:**
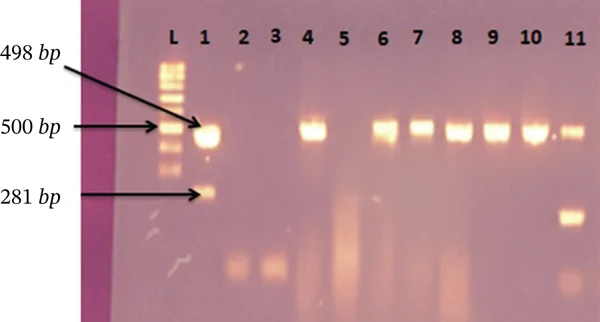
Agarose gel electrophoresis for the multiplex PCR‐amplified products of *K. pneumoniae bla_KPC_* and *bla_OXA-48_* genes. L: DNA ladder (100 bp); Lane 1: control positive for *bla_KPC_* (498 bp) and *bla_OXA-48_* (281 bp) genes; Lane 2: control negative; Lanes 3 and 5: negative samples for both genes; Lanes 4, 6, 7, 8, 9, and 10: positive samples for *bla_KPC_* gene only; and Lane 11: positive sample for both genes.

**Figure 2:**
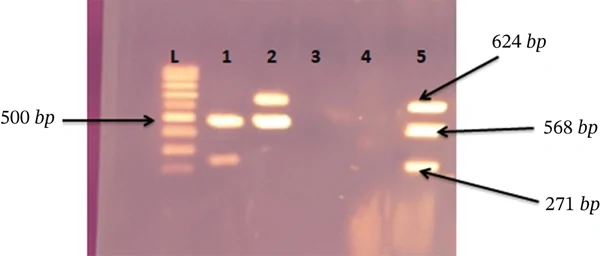
Agarose gel electrophoresis for the multiplex PCR‐amplified products of *K. pneumoniae bla_SPM_*, *bla_NDM_*, and *bla_IMP_* genes. L: DNA ladder (100 bp); Lane 5: control positive for *bla_SPM_* (271 bp), *bla_NDM_* (624 bp), and *bla_IMP_* (568 bp) genes; Lane 4: control negative; Lane 3: negative sample for all genes; Lane 2: positive sample for *bla_NDM_* and *bla_IMP_* genes; and Lane 1: positive sample for *bla_SPM_* and *bla_IMP_* genes.

Out of the 50 samples tested for carbapenemase genes, 6 (12%) were negative for all tested genes, whereas 44 (88%) were positive for at least one carbapenemase gene. Among the positive samples, 28 (56.0% of total isolates, 63.6% of gene‐positive isolates) harbored more than one gene, and 16 (32.0% of total isolates, 36.4% of gene‐positive isolates) were positive for a single gene. The overall frequency of carbapenemase genes was as follows: *bla_KPC_* in 36 isolates (72%), *bla_OXA-48_* in 5 isolates (10%), *bla_SPM_* in 19 isolates (38%), *bla_NDM_* in 21 isolates (42%), and *bla_IMP_* in 14 isolates (28%) (Table 4, Figures 1 and 2). Among the 28 isolates carrying multiple genes, 12 isolates had two genes, 9 isolates had three genes, and 7 isolates had four genes (Table 5). The most common combinations were four genes (*bla_KPC_* + *bla_SPM_* + *bla_NDM_* + *bla_IMP_*) in 6 isolates (21.4%), two genes (*bla_SPM_* + *bla_NDM_*) in 4 isolates (14.3%), and three genes (*bla_KPC_* + *bla_SPM_* + *bla_NDM_*) in 4 isolates (14.3%).

**Table 4:** Distribution of carbapenemase genes detected in *Klebsiella pneumoniae* isolates.

Category/gene target	Number of isolates	Percentage of total sample (*N* = 50)	Percentage of positive sample (*n* = 44)
Overall gene status			
Negative for all tested genes	6	12.0%	—
Positive for carbapenemase gene	44	88.0%	100.0%
Carriage pattern (among positives)			
Single gene carriage	16	32.0%	36.4%
Multiple gene codetection	28	56.0%	63.6%
Individual carbapenemase genes detected			
*bla_KPC_*	36	72.0%	81.8%
*bla_NDM_*	21	42.0%	47.7%
*bla_SPM_*	19	38.0%	43.2%
*bla_IMP_*	14	28.0%	31.8%
*bla_OXA-48_*	5	10.0%	11.4%

**Table 5:** Patterns of carbapenemase gene coexistence.

Codetection type	Gene combination	Number of isolates (*n*)	Percentage (%)
Two genes (*n* = 12)	*bla_SPM_* + *bla_NDM_*	4	14.3%
	*bla_KPC_* + *bla_OXA-48_*	3	10.7%
	*bla_KPC_* + *bla_NDM_*	2	7.1%
	*bla_KPC_* + *bla_IMP_*	2	7.1%
	*bla_SPM_* + *bla_IMP_*	1	3.6%

Three genes (*n* = 9)	*bla_KPC_* + *bla_SPM_* + *bla_NDM_*	4	14.3%
	*bla_KPC_* + *bla_SPM_* + *bla_IMP_*	2	7.1%
	*bla_SPM_* + *bla_NDM_* + *bla_IMP_*	1	3.6%
	*bla_KPC_* + *bla_NDM_* + *bla_IMP_*	1	3.6%
	*bla_KPC_* + *bla_OXA-48_* + *bla_IMP_*	1	3.6%

Four genes (*n* = 7)	*bla_KPC_* + *bla_SPM_* + *bla_NDM_* + *bla_IMP_*	6	21.4%
	*bla_KPC_* + *bla_OXA-48_* + *bla_SPM_* + *bla_NDM_*	1	3.6%

Total	All codetecting isolates	28	100.0%

The distribution of carbapenemase genes across infection sites, gender, age groups, and prior antibiotic exposure showed some variability, but statistically significant associations were limited. No significant association was detected between the presence of *bla_KPC_*, *bla_OXA-48_*, *bla_SPM_*, or *bla_IMP_* and the site of infection (all p > 0.05), whereas a statistically significant association was observed for *bla_NDM_* (*p* = 0.039) (Table 6). Likewise, there was no statistically significant association between any of these carbapenemase genes and patient age group (all *p* > 0.05) (Table 7). No significant associations were observed between gender and the presence of *bla_KPC_*, *bla_OXA-48_*, *bla_SPM_*, *bla_NDM_*, or *bla_IMP_* (all *p* > 0.05) (Table 8). In contrast, prior antibiotic exposure showed a statistically significant association with the presence of *bla_SPM_* (p ≈ 0.02), whereas no significant association was found between prior antibiotic use and *bla_KPC_*, *bla_OXA-48_*, *bla_NDM_*, or *bla_IMP_* (all *p* > 0.05) (Table 9).

**Table 6:** Distribution of carbapenemase genes by infection site.

Carbapenemase genes	Infection site						Total (%)	*p*
	Urinary tract infection (%)	Gastroenteritis (%)	Genital tract infection (%)	Respiratory tract infection (%)	Wound infection (%)	Blood infection (%)		
*bla_KPC_*	17 (77%)	0 (0%)	2 (66%)	5 (83%)	8 (61%)	4 (100%)	36 (72%)	*0.149*
*bla_OXA-48_*	2 (9%)	0 (0%)	0 (0%)	2 (33%)	1 (7%)	0 (0%)	5 (10%)	*0.450*
*bla_SPM_*	10 (46%)	2 (100%)	0 (0%)	2 (33%)	3 (23%)	2 (50%)	19 (38%)	*0.210*
*bla_NDM_*	11 (50%)	2 (100%)	0 (0%)	0 (0%)	5 (38%)	3 (75%)	21 (42%)	*0.039*
*bla_IMP_*	8 (36%)	0 (0%)	0 (0%)	3 (50%)	2 (15%)	1 (25%)	14 (28%)	*0.393*

**Table 7:** Distribution of carbapenem‐resistant genes by age group.

Carbapenemase genes	Age groups					Total (%)	*p*
	< 15 years (%)	16‐40 years (%)	41–65 years (%)	66–90 years (%)	> 90 years (%)		
*bla_KPC_*	6 (75%)	10 (71%)	10 (71%)	10 (76%)	0 (0%)	36 (72%)	*0.60*
*bla_OXA-48_*	0 (0%)	2 (14%)	2 (14%)	1 (7%)	0 (0%)	5 (10%)	*0.80*
*bla_SPM_*	4 (50%)	3 (21%)	6 (42%)	6 (46%)	0 (0%)	19 (38%)	*0.52*
*bla_NDM_*	5 (62%)	5 (35%)	5 (35%)	6 (46%)	0 (0%)	21 (42%)	*0.62*
*bla_IMP_*	2 (25%)	3 (21%)	5 (35%)	4 (30%)	0(0%)	14 (28%)	*0.88*

**Table 8:** Distribution of carbapenemase genes by gender.

Carbapenemase genes	Gender		Total (%)	*p*
	Female (%)	Male (%)		
*bla_KPC_*	14 (73%)	22 (70%)	36 (72%)	*1.000*
*bla_OXA-48_*	1 (5%)	4 (12%)	5 (10%)	*0.637*
*bla_SPM_*	6 (31%)	13 (41%)	19 (38%)	*0.556*
*bla_NDM_*	9 (47%)	12 (38%)	21 (42%)	*0.570*
*bla_IMP_*	4 (21%)	10 (32%)	14 (28%)	*0.522*

**Table 9:** Distribution of carbapenemase genes by antibiotic exposure.

Carbapenemase genes	Antibiotic Exposure		Total (%)	*p*
	Exposed (%)	Nonexposed (%)		
*bla_KPC_*	31 (75%)	5 (55%)	36 (72%)	*0.422*
*bla_OXA-48_*	4 (9%)	1 (11%)	5 (10%)	*1.000*
*bla_SPM_*	12 (29%)	7 (77%)	19 (38%)	*0.020*
*bla_NDM_*	15 (36%)	6 (66%)	21 (42%)	*0.200*
*bla_IMP_*	9 (21%)	5 (55%)	14 (28%)	*0.105*

## 4. Discussion

This study analyzed 50 CRKP isolates and found a high prevalence of carbapenemase genes. Out of the 50 samples tested, 6 (12%) were negative for all tested genes, whereas 44 (88%) carried at least one carbapenemase gene. This overall positive rate (88%) is higher than the average reported across Africa by Sisay et al. [9]. This difference is likely because we specifically tested resistant samples from hospital patients in Khartoum, rather than general community samples.

In the present study, a panel of five carbapenemase genes (*bla_NDM_*, *bla_KPC_*, *bla_OXA-48_*, *bla_IMP_*, and *bla_SPM_*) was selected to provide a comprehensive molecular screening covering Class A, B, and D carbapenemases [1, 2]. Although *bla_SPM_* is less frequently reported compared with *bla_NDM_* or *bla_OXA-48_* in regional isolates [9, 10], its inclusion was justified to establish baseline epidemiological surveillance for metallo‐*β*‐lactamases capable of horizontal gene transfer across gram‐negative pathogens [4, 11]. Remarkably, *bla_SPM_* exhibited a statistically significant association with prior antibiotic exposure (*p* = 0.020), highlighting the role of selective antimicrobial pressure in harboring less common resistance determinants within healthcare settings [12, 13].

The most common gene was *bla_KPC_* (72%), followed by *bla_NDM_* (42%), *bla_SPM_* (38%), *bla_IMP_* (28%), and *bla_OXA-48_* (10%). This distribution is consistent with global reports showing that *bla_KPC_* and *bla_NDM_* are the most widespread carbapenemases worldwide [14]. Similar patterns and regional variations have also been reported by Ubeda et al. [15] and Albasha et al. [10]. The high prevalence of *bla_KPC_* in our setting may be related to local antibiotic use and hospital‐specific selective pressure, as suggested by Wang et al. [12]. Interestingly, earlier studies in Sudan, such as Abdel Hamid et al. [16] at Soba University Hospital, found *bla_NDM_* and *bla_OXA-48_* to be the main genes. Finding *bla_KPC_* as the top gene in our study shows how resistance patterns are changing in local hospitals over time, matching global trends described by Bonomo et al. [1].

A key finding was the frequent presence of multiple carbapenemase genes within the same isolate. In this study, 28 out of 44 carbapenemase‐positive isolates (63.6%) carried more than one gene, whereas 16 isolates (36.4%) had only a single gene. Similar co‐occurrence has been reported by Takahashi et al. [17]. The presence of multiple resistance genes makes treatment more difficult and is often linked to resistance to many antibiotics. Plasmid‐mediated gene transfer likely contributes to the spread of these genes in healthcare settings, especially where antibiotic use is high [11]. Our results also build on recent DNA sequencing work in Sudan by Osman et al. [18], who showed high antibiotic resistance in local bacteria. Although recent WGS surveys in Sudan have reported an overall carbapenem resistance rate of approximately 41% among clinical isolates, our study advances the local epidemiological understanding by specifically detailing the high‐frequency codetection patterns and multigene combinations driving this resistance in a preselected highly resistant cohort.

Interestingly, 6 isolates (12%) were resistant to carbapenems but did not carry any of the five tested genes (*bla_KPC_*, *bla_NDM_*, *bla_OXA-48_*, *bla_IMP_*, or *bla_SPM_*). As explained by Nordmann and Poirel [19], resistance is not always caused by carbapenemase genes. Other mechanisms, such as blocked cell membrane channels or active pumps that push the drug out, can also make bacteria resistant.

No significant association was found between specific carbapenemase genes and the type of clinical specimen. This finding agrees with Ubeda et al. [15] and suggests that gene distribution is influenced more by hospital and patient‐related factors than by infection site. Similarly, no significant relationship was observed between age group and the presence of any carbapenemase gene. The detection of resistant isolates across all age groups highlights the widespread nature of this problem.

In addition, no significant association was found between gender and the presence of *bla_KPC_*, *bla_OXA-48_*, *bla_SPM_*, *bla_NDM_*, or *bla_IMP_*. This suggests that demographic factors such as age and sex are not major determinants of carbapenemase distribution in our setting. Other factors, including hospital exposure, underlying conditions, and antibiotic use, are likely more important.

Although a statistically significant association was found between prior antibiotic exposure and *bla_SPM_* (p ≈ 0.020), this result should be interpreted with caution. Surprisingly, *bla_SPM_* was more common in patients without prior antibiotic use (77%) than in those with exposure (29%). This unexpected finding is likely due to the small size of the unexposed group (*n* = 9). Still, these findings highlight the need for proper antibiotic stewardship in hospitals.

This study has several limitations that should be acknowledged. First, the sample size was relatively modest (*n* = 50) and collected from a single geographical region, which may limit generalizability to all national healthcare settings. Second, PCR‐amplified products were not confirmed by Sanger sequencing or restriction digestion, leaving a small possibility of nonspecific amplifications. Finally, phenotypic mechanisms such as porin expression and efflux pump activity were not functionally evaluated in gene‐negative resistant strains.

## 5. Conclusion and Recommendation

This study found a high rate of carbapenemase‐producing *K. pneumoniae* among carbapenem‐resistant isolates from different clinical specimens. Most isolates carried at least one carbapenemase gene, with *bla_KPC_* being the most common, followed by *bla_NDM_, bla_SPM_*, *bla_IMP_*, and *bla_OXA-48_*. Many isolates contained more than one carbapenemase gene, showing that resistance in these bacteria is complex.

No significant relationship was found between carbapenemase genes and infection site, age group, or gender. This indicates that carbapenem‐resistant *K. pneumoniae* can affect patients of all ages and both sexes and can be isolated from different body sites. However, prior antibiotic use was significantly associated with the presence of *bla_SPM_*, suggesting that antibiotic exposure plays a role in selecting certain resistance genes.

These findings highlight the need for strict infection control measures and improved antibiotic stewardship to reduce the spread of carbapenem‐resistant *K. pneumoniae*. Further studies with larger sample sizes are recommended to better understand resistance patterns and support effective prevention and treatment strategies.

Given the dominance of *bla_KPC_* and multigene co‐producers, routine phenotypic confirmation and active molecular screening for *bla_KPC_* producers upon hospital admission are highly recommended. These targeted infection control interventions are critical to interrupt the nosocomial transmission of these highly resistant clones.

## Funding

No funding was received for this manuscript.

## Conflicts of Interest

The authors declare no conflicts of interest.

## Data Availability

The data that support the findings of this study are available from the corresponding author upon reasonable request.
